# AHG-YOLO: multi-category detection for occluded pear fruits in complex orchard scenes

**DOI:** 10.3389/fpls.2025.1580325

**Published:** 2025-05-23

**Authors:** Na Ma, Yile Sun, Chenfei Li, Zonglin Liu, Haiyan Song

**Affiliations:** ^1^ College of Agricultural Engineering, Shanxi Agricultural University, Jinzhong, China; ^2^ Dryland Farm Machinery Key Technology and Equipment Key Laboratory of Shanxi Province, Jinzhong, China; ^3^ College of Information Science and Engineering, Shanxi Agricultural University, Jinzhong, China

**Keywords:** YOLOv11, pear fruits, object detection, ADown, group convolution, GIoU

## Abstract

**Introduction:**

To achieve fast detection of pear fruits in natural pear orchards and optimize path planning for harvesting robots, this study proposes the AHG-YOLO model for multi-category detection of pear fruit occlusion in complex orchard environments.

**Methods:**

Using the Red Delicious pear as the research object, the pears are classified into three categories based on different occlusion statuses: non-occluded fruits (NO), fruits occluded by leaves/branches (OBL), and fruits in close contact with other fruits but not obstructed by leaves/branches (FCC). The YOLOv11n model is used as the base model for a lightweight design. First, the sampling method in the backbone and neck networks is replaced with ADown downsampling to capture higher-level image features, reducing floating-point operations and computational complexity. Next, shared weight parameters are introduced in the head network, and group convolution is applied to achieve a lightweight detection head. Finally, the boundary box loss function is changed to Generalized Intersection over Union (GIoU), improving the model’s convergence speed and further enhancing detection performance.

**Results:**

Experimental results show that the AHG-YOLO model achieves 93.5% (FCC), 95.3% (NO), and 93.4% (OBL) in AP, with an mAP@0.5 of 94.1% across all categories. Compared to the base YOLOv11n network, precision, recall, mAP@0.5, and mAP@0.5:0.95 are improved by 2.5%, 3.6%, 2.3%, and 2.6%, respectively. The model size is only 5.1MB, with a 16.9% reduction in the number of parameters.

**Discussion:**

The improved model demonstrates enhanced suitability for deployment on pear-harvesting embedded devices, providing technical support for the path planning of fruit-picking robotic arms.

## 1 Introduction

The pear is a nutrient-rich fruit with high economic and nutritional value, widely cultivated around the world (Seo et al., 2022). China has been the largest producer and consumer of fruits globally, with orchard area and production continuously increasing (Zhang et al., 2024). Fruit harvesting has become one of the most time-consuming and labor-intensive processes in fruit production (Vrochidou et al., 2022). In the complex orchard environment, accurate fruit detection is essential for achieving orchard automation and intelligent management (Bharad and Khanpara, 2024; Chen et al., 2024). Currently, pear harvesting mainly relies on manual labor, which is inefficient. Additionally, with the aging population and labor shortages, the cost of manual harvesting is rising, making the automation of pear fruit harvesting an urgent problem to address. In recent years, researchers have been focusing on mechanized and intelligent fruit harvesting technologies (Parsa et al., 2024). However, in the unstructured environment of orchards, fruits are often occluded by branches and leaves, and their growth orientations vary, which affects the accuracy of detection and localization, posing significant challenges to automated fruit harvesting (Tang et al., 2023).

Traditional image processing methods for detecting fruit targets require manually designed features, such as color features, shape features, and texture features (Liu and Liu, 2024). These methods then combine machine learning algorithms with the manually designed features to detect fruits, but detection accuracy can be easily affected by subjective human factors, and detection efficiency is low (Dhanya et al., 2022).

In recent years, with the development of image processors (GPUs) and deep learning technologies, significant progress has been made in the field of object detection. Algorithms such as Faster R-CNN (Ren et al., 2016) and SSD (Liu et al., 2016) have demonstrated excellent performance in general tasks. However, these methods still face challenges in real-time processing or small object detection. The YOLO series such as YOLOv5 (Horvat et al., 2022), YOLOv6 (Li et al., 2022), YOLOv7 (Wang et al., 2023), YOLOv8 (Sohan et al., 2024), YOLOv9 (Wang et al., 2024), YOLOv10 (Alif and Hussain, 2024), YOLOv11 (Khanam and Hussain, 2024) has shown improvements in both speed and accuracy, leading many researchers to utilize YOLO algorithms for fruit detection research. Liu et al. (2024) proposed a new lightweight apple detection algorithm called Faster-YOLO-AP based on YOLOv8. The results showed that Faster-YOLO-AP reduced its parameters and FLOPs to 0.66 M and 2.29G, respectively, with an mAP@0.5:0.95 of 84.12%. Zhu et al. (2024) introduced an improved lightweight YOLO model (YOLO-LM) based on YOLOv7-tiny for detecting the maturity of tea oil fruits. The precision, recall, mAP@0.5, parameters, FLOPs, and model size were 93.96%, 93.32%, 93.18%, 10.17 million, 19.46 G, and 19.82 MB, respectively. Wei et al. (2024) proposed a lightweight tomato maturity detection model named GFS-YOLOv11, which improved precision, recall, mAP@0.5, and mAP@0.5:0.95 by 5.8%, 4.9%, 6.2%, and 5.5%, respectively. Tang et al., 2024 addressed the issue of low detection accuracy and limited generalization capabilities for large non-green mature citrus fruits under different ripeness levels and varieties, proposing a lightweight real-time detection model for unstructured environments—YOLOC-tiny. Sun et al. (2023) focused on efficient pear fruit detection in complex orchard environments and proposed an effective YOLOv5-based model—YOLO-P—for fast and accurate pear fruit detection. However, in complex, unstructured orchard environments, factors such as varying lighting conditions, occlusions, and fruit overlaps still affect recognition accuracy and generalization capabilities. Additionally, existing models often suffer from high computational complexity and excessive parameters, making them difficult to deploy on resource-constrained mobile or embedded devices. To address these challenges, researchers have been committed to designing high-precision, fast detection models that meet the requirements for real-time harvesting.

Current research on pear fruit detection has made some progress. Ren et al. (2023) proposed the YOLO-GEW network based on YOLOv8 for detecting “Yulu Xiang” pear fruits in unstructured environments, achieving a 5.38% improvement in AP. Zhao et al. (2024) developed a high-order deformation-aware multi-object search network (HDMNet) based on YOLOv8 for pear fruit detection, with a detection accuracy of 93.6% in mAP@0.5 and 70.2% in mAP@0.75. Lu et al. (2023) introduced the ODL Net algorithm for detecting small green pear fruits, achieving detection accuracies of 56.2% and 65.1% before and after fruit thinning, respectively. Shi et al. (2024) proposed an improved model, YOLOv9s-Pear, based on the lightweight YOLOv9s model, enhancing the accuracy and efficiency of red-skinned young pear recognition. The model achieved precision, recall, and AP rates of 97.1%, 97%, and 99.1%, respectively. The aforementioned studies primarily focus on single pear fruit detection during maturity or young fruit stages. However, in practical harvesting scenarios, considerations such as robotic arm picking strategies and path planning are also crucial (Wang et al., 2020). The picking strategy and path planning of robotic arms are closely related to the fruit’s growth position. Detailed classification of fruit location information enables harvesting robots to adapt flexibly to varying environmental conditions, dynamically adjusting path planning and grasping strategies to ensure efficient and precise harvesting operations. This enhances the system’s flexibility and robustness in complex scenarios (Nan et al., 2023).

Based on the aforementioned background, this paper proposes a lightweight intelligent pear orchard fruit detection method, AHG-YOLO, using YOLOv11n as the base model. First, the traditional sampling method in the YOLOv11n backbone and neck networks is replaced with ADown to reduce computational complexity while improving detection accuracy. Next, a new detection head structure is developed using the “shared” concept and group convolution to further lighten the model without compromising detection performance. Finally, the CIoU loss function in YOLOv11n is replaced with GIoU to enhance the model’s accuracy and fitting capability. The improved model not only maintains high recognition accuracy but also reduces the model size and computational cost, making it easier to deploy on mobile devices. This provides technical support for optimizing robotic picking paths and meets the demands of intelligent harvesting in pear orchards.

## 2 Material and methodology

### 2.1 Image collection

**Figure 1:**
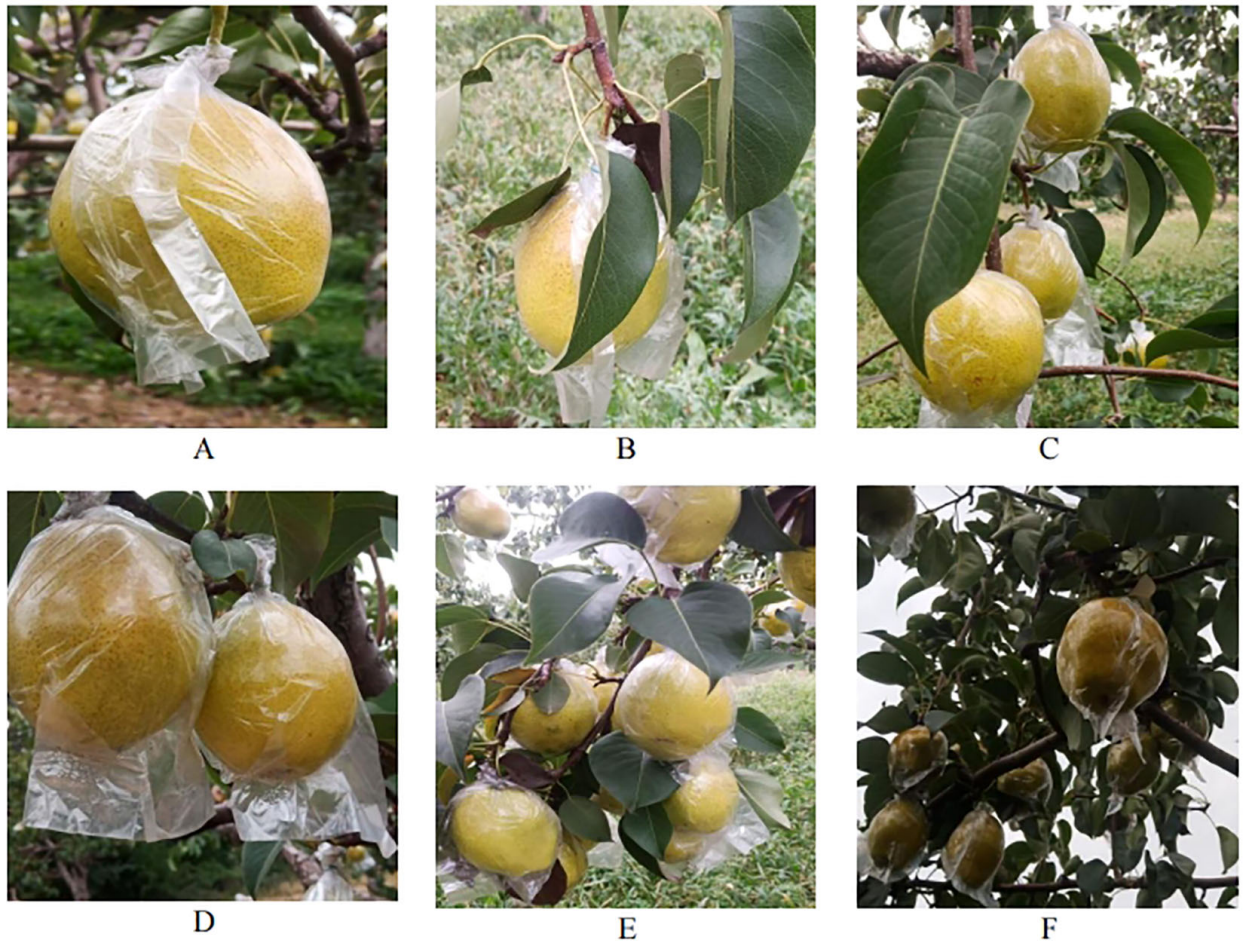
Sample images. **(A)** Single fruit; **(B)** Single fruit + leaf obstruction; **(C)** Multiple fruits + branch and leaf obstruction; **(D)** Overlapping fruits; **(E)** Backlight + dense fruits; **(F)** Cloudy weather + dense fruits.

The Hongxiangsu pear, known as the “king of all fruits,” is a hybrid descendant of the Korla fragrant pear and the goose pear, and is a late-maturing, storage-resistant red-skinned pear variety. The fruit is spindle-shaped, weighing an average of 220 grams, with a maximum weight of 500 grams. The fruit surface is smooth and clean, with a bright red color. The flesh is white, fine-grained, sweet, and aromatic, with medicinal properties such as clearing heat, moisturizing the lungs, relieving cough, quenching thirst, and aiding in alcohol detoxification. It also has health benefits for conditions such as hypertension, high cholesterol, and arteriosclerosis. This study focuses on the Hongxiangsu pear, and data was collected from the Modern Agricultural Industry Technology System Demonstration Base of the Fruit Tree Research Institute at Shanxi Agricultural University, located in Taigu District, Jinzhong City, Shanxi Province (112°32’E, 37°23’N). Considering that the harvesting robotic arm needs to adapt to the complex environment of the orchard during the harvesting process, pear images were captured from various angles, distances, and time periods using a Vivo Z3i smartphone. A total of 1,000 pear images were collected, and unusable images were filtered out, leaving 734 usable images. The complex orchard environment includes scenarios such as single fruit, multiple fruits, cloudy weather, overlapping fruits, and branches and leaves obstructing the view. Some sample images are shown in **Figure 1**.

### 2.2 Data augmentation

**Figure 2:**
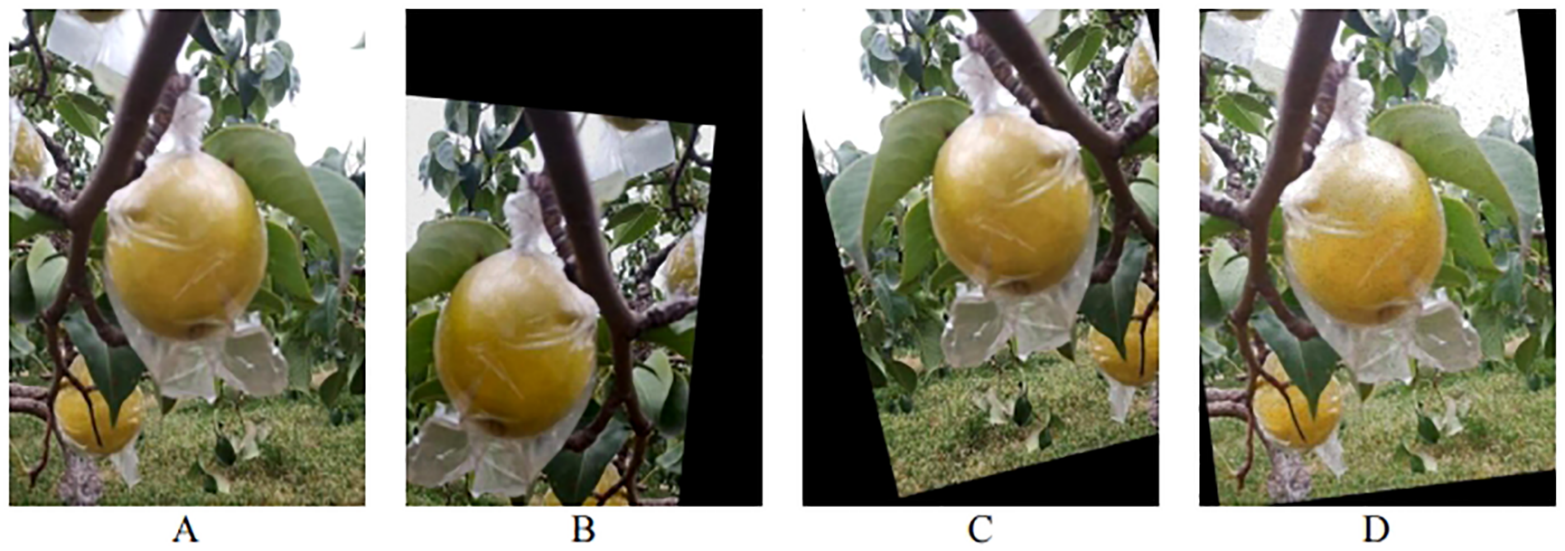
Image data augmentation examples. **(A)** Original image; **(B)** Brightness Adjustment + Rotation; **(C)** Image Sharpening + Rotation; **(D)** Salt-and-Pepper Noise + Rotation.

To improve the robustness and generalization ability of the pear object detection model, image sample data needs to be augmented. In this study, various augmentation techniques, including adding salt-and-pepper noise, image sharpening, affine transformation, and brightness adjustment, are randomly combined to enhance the images. After data augmentation, the total number of pear samples is 2936. The dataset is split into training set (2055 images), validation set (293 images), and test set (588 images) with a ratio of 7:1:2. Some of the augmented data samples are shown in **Figure 2**.

### 2.3 Dataset construction

**Figure 3:**
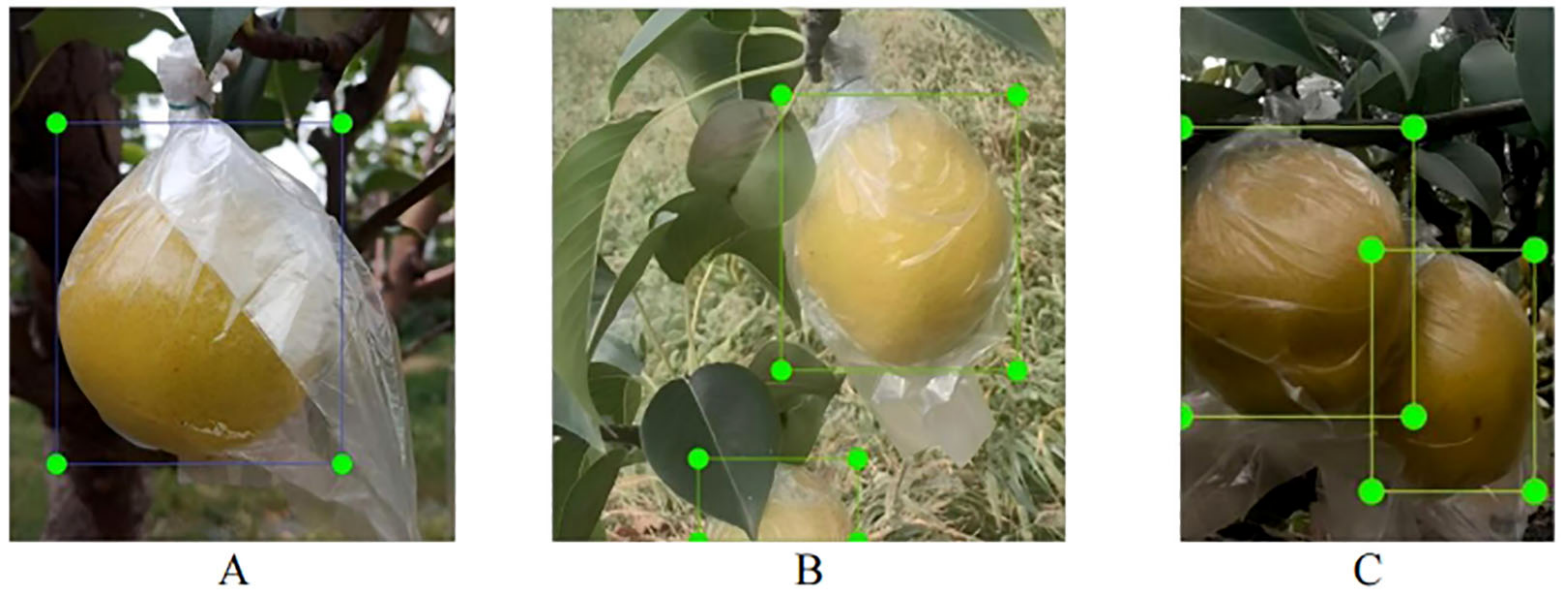
Annotated Example of Pear Fruit Classification. **(A)** Fruits that are not obstructed (referred to as NO); **(B)** fruits that are occluded by branches or leaves (referred to as OBL); **(C)** fruits that are close contact with other fruits but are not occluded by branches or leaves (referred to as FCC).

In natural environments, pear fruits are often obstructed by leaves or branches, and fruits can occlude each other, posing significant challenges for robotic harvesting. To improve harvesting efficiency, the harvesting robot can adopt different strategies when encountering pears in various scenarios during the harvesting process. For example, for an unobstructed target, path planning is relatively simple, and conventional path planning and grabbing tasks can be directly applied. When the target is partially occluded, path planning needs to consider how to navigate around the obstruction or adjust the grabbing angle. In environments with dense fruits, where occlusion and overlap of multiple fruits are concerns, multi-object path planning algorithms can be used to devise the optimal path (Gao, 2023; Yang et al., 2022). Therefore, based on the growth loci characteristics, the fruits are systematically categorized into three distinct classes in this study. The schematic of the three categories of pears is shown in **Figure 3**. The first class represents fruits that are not obstructed (referred to as NO). The second class represents fruits that are occluded by branches or leaves (referred to as OBL). The third class represents fruits that are in close contact with other fruits but are not occluded by branches or leaves (referred to as FCC). This classification standard is based on the classification criteria proposed by Nan et al. (2023) for pitaya fruits.

The pear fruits in the images were annotated using rectangular bounding boxes in Labeling (Tzutalin, 2015) software, categorized into three classes (NO, OBL, and FCC) according to the predefined classification criteria. The annotations were formatted in YOLO style and ultimately saved as.txt files. Upon completion of the annotation process, the distribution of different categories across the final training set, validation set, and test set is shown in **Table 1**.

**Table 1:** Distribution of data in different categories.

Classes	Bounding boxes number in train	Bounding boxes number in val	Bounding boxes number in test
NO	3035	381	865
OBL	7731	1016	2256
FCC	3532	512	1039

### 2.4 AHG-YOLO

**Figure 4:**
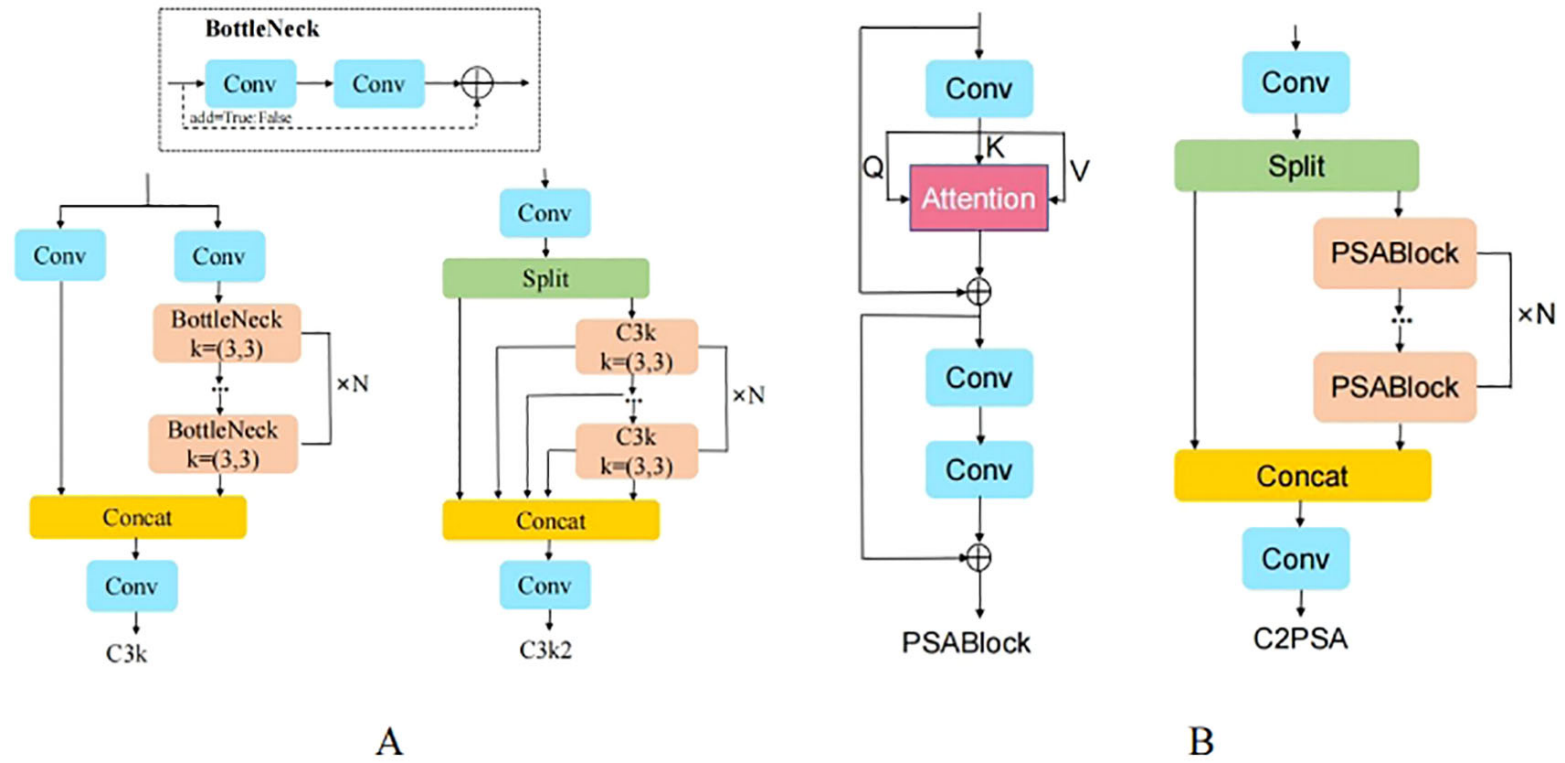
Module Structure. **(A)** C3k2; **(B)** C2PSA and PSABlock.

**Figure 5:**
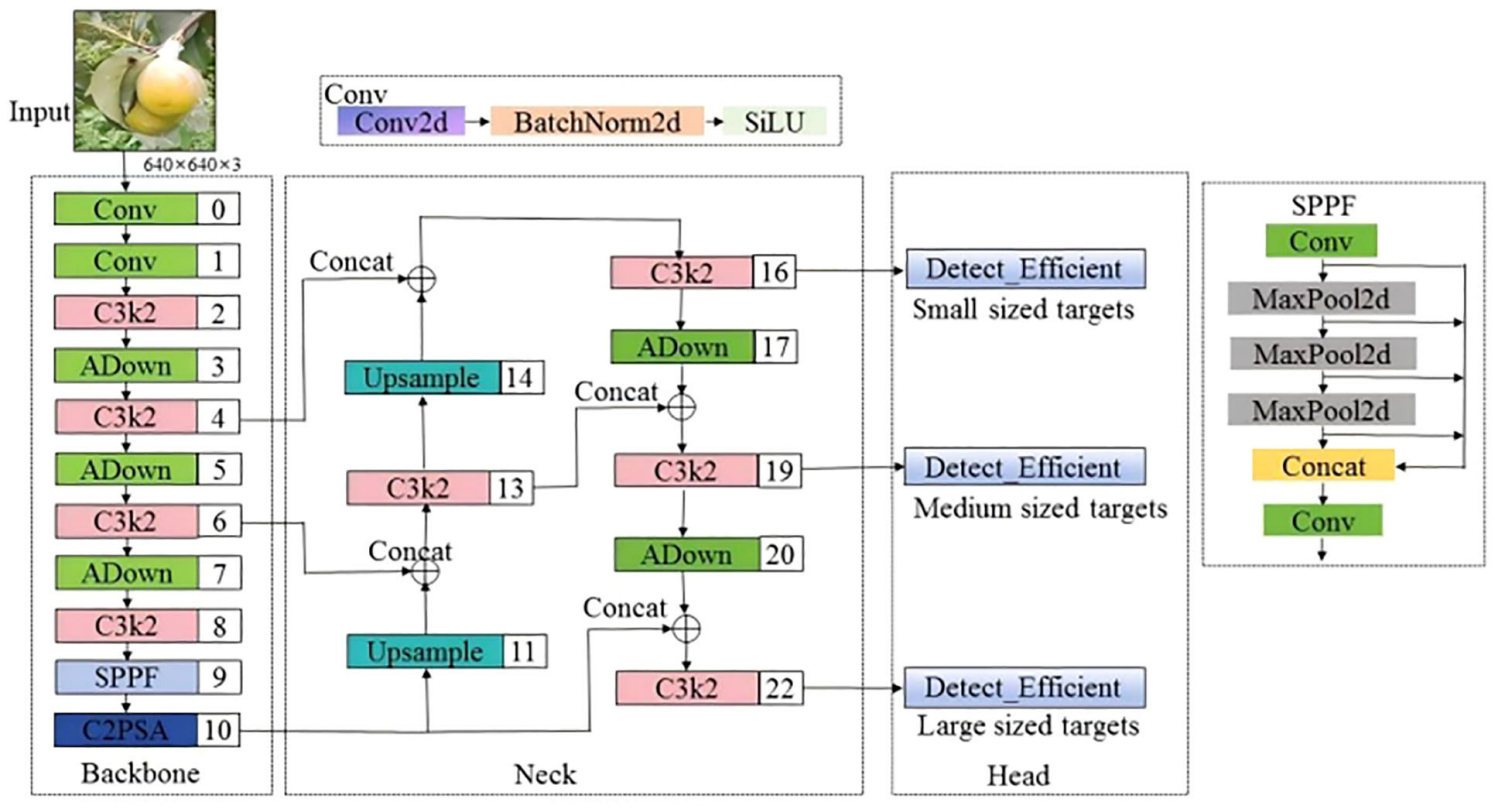
The AHG-YOLO network structure.

The YOLOv11 network introduces two innovative modules, C3k2 and C2PSA, as shown in **Figure 4**, which further enhance the network’s accuracy and speed. However, in unstructured environments such as orchards, when fruits are severely occluded, overlapping, or when the fruit targets are small, the YOLOv11 network is prone to missing or misdetecting targets. To enhance the accuracy and robustness of pear detection algorithms in unstructured environments, this paper improves the YOLOv11n model. The architecture of the improved model is shown in **Figure 5**. First, in both the backbone and head networks, the downsampling method is replaced with ADown (Wang et al., 2024), enabling the model to capture image features at higher levels, enhancing the feature extraction capability of the network and reducing computational complexity. Then, a lightweight detection head, Detect_Efficient, is designed, which further reduces the computational load by sharing the detection head and incorporating group convolution, while improving the network’s feature extraction capacity. Finally, the CIou loss function of YOLOv11 is replaced with GIoU (Jiang et al., 2023), which reduces the impact of low-quality samples and accelerating the convergence of the network model. The proposed improvements are named AHG-YOLO, derived from the first letters of the three improvement methods: ADown, Head, and GIoU. The AHG-YOLO model effectively improves pear detection performance and better adapts to the detection needs of small targets, occlusion, and fruit overlap in the complex natural environment of pear orchards.

#### 2.4.1 ADown

The ADown module in YOLOv9 is a convolutional block for downsampling in object detection tasks. As an innovative feature in YOLOv9, it provides an effective downsampling solution for real-time object detection models, combining lightweight design and flexibility. In deep learning models, downsampling is a common technique used to reduce the spatial dimensions of feature maps, enabling the model to capture image features at higher levels while reducing computational load. The ADown module is specifically designed to perform this operation efficiently with minimal impact on performance.

**Figure 6:**
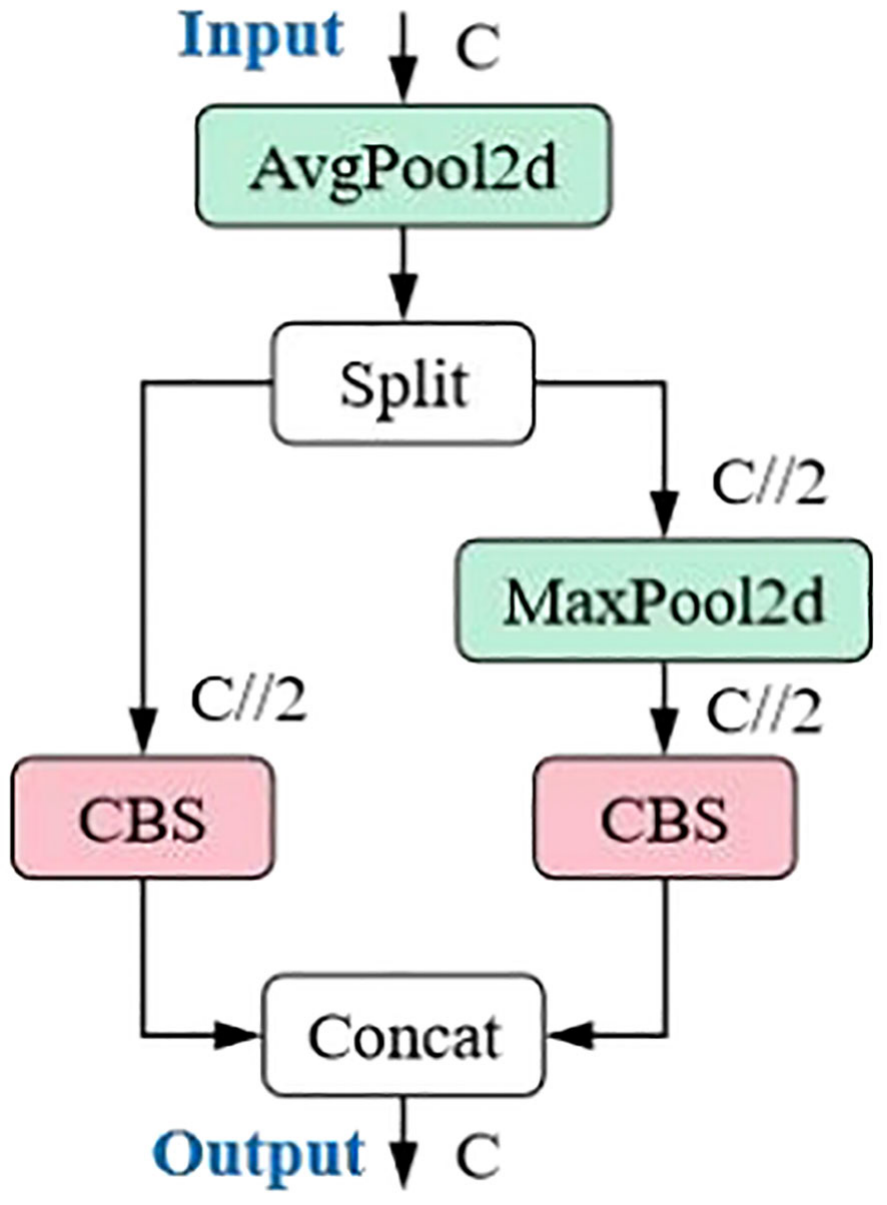
A down network structure.

The main features of the ADown module are as follows: (1) Lightweight design: The ADown module reduces the number of parameters, which lowers the model’s complexity and enhances operational efficiency, especially in resource-constrained environments. (2) Information preservation: Although ADown reduces the spatial resolution of feature maps, its design ensures that as much image information as possible is retained, allowing the model to perform more accurate object detection. (3) Learnable capabilities: The ADown module is designed to be learnable, meaning it can be adjusted according to different data scenarios to optimize performance. (4) Improved accuracy: Some studies suggest that using the ADown module not only reduces the model size but also improves object detection accuracy. (5) Flexibility: The ADown module can be integrated into both the backbone and head of YOLOv9, offering various configuration options to suit different enhancement strategies. (6) Combination with other techniques: The ADown module can be combined with other enhancement techniques, such as the HWD (Wavelet Downsampling) module, to further boost performance. The ADown network structure is shown in **Figure 6**.

By introducing the ADown module into YOLOv9, a significant reduction in the number of parameters can be achieved, while maintaining or even improving object detection accuracy. Consequently, this study explores the integration of the ADown module into the YOLOv11 network structure to further enhance detection performance.

#### 2.4.2 Detection head re-design

**Figure 7:**
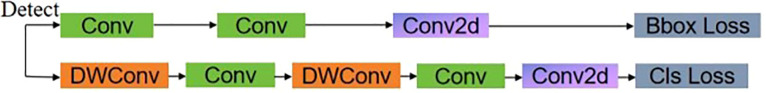
YOLOv11 detection head.

The detection decoupled head structure of YOLOv11n is shown in **Figure 7**. The extracted feature map is passed through two branches. One branch undergoes two 3×3 convolutions followed by a 1×1 convolution, while the other branch undergoes two depth wise separable convolutions (DWConv), two 3×3 convolutions, and a 1×1 convolution. These branches are used to independently predict the bounding box loss and the classification function.

In YOLOv11, there are three of the aforementioned decoupled head structures, which perform detection on large, medium, and small feature maps. However, 3×3 convolutions, while increasing the channel depth, lead to a significant increase in the number of parameters and floating-point operations (Shafiq and Gu, 2022). Therefore, this study aims to implement a lightweight design for YOLOv11’s detection head while maintaining detection accuracy:

(1) Introducing Group Convolutions to Replace 3×3 Convolutions.

Group Convolution is a convolution technique used in deep learning primarily to reduce computation and parameter quantities while enhancing the model’s representational power. Group convolution works by dividing the input feature map and convolution kernels into several groups. Each group performs its convolution operation independently, and the results are then merged. This process reduces the computation and parameter quantities while maintaining the same output size.

In traditional convolution operations, the convolution is applied across every channel of the input feature map. Assuming the input feature map has dimensions ${\text{C}}_{\text{in}}\times \text{H}\times$ W ( ${\text{C}}_{\text{in}}$ is the number of input channels, H is the feature map height, and W is the feature map width), and the convolution kernel has dimensions ${\text{ C}}_{\text{out}}\times {\text{C}}_{\text{in}}\times \text{k}\times \text{k}$ ( ${\text{C}}_{\text{out}}$ is the number of output channels and k×k is the spatial dimension of the kernel), the computation for a single convolution operation is: ${\text{ C}}_{\text{out}}\times {\text{C}}_{\text{in}}\times \text{k}\times \text{k}\times \text{H}\times \text{W}$ .

In group convolution, the input channels are divided into g groups, and independent convolution operations are performed within each group. In this case, for each group, the number of input channels becomes ${\text{C}}_{\text{in}}$ /g, and the computation becomes: ${\text{ C}}_{\text{out}}\times {\text{C}}_{\text{in}}/\text{g}\times \text{k}\times \text{k}\times \text{H}\times \text{W}$ .

Group convolution can greatly reduce the number of parameters, enhance the model’s representational power, and avoid overfitting. Therefore, the 3×3 convolutions in the detection head are replaced with group convolutions.

(2) Shared Convolution Parameters.

**Figure 8:**
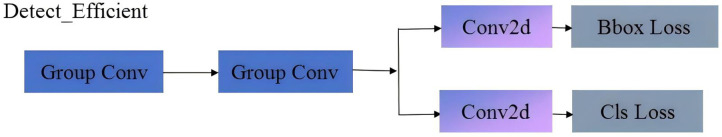
Detect_efficient structure.

To further reduce the parameters and computation of the detection head, the two branch inputs of the detection head share two group convolutions, named Detect_Efficient, with the structure shown in **Figure 8**. By sharing the same convolution kernel weights during loss calculation, redundant computation of similar feature maps is avoided, which further reduces the computation and effectively improves computational efficiency, accelerating the entire model inference process.

#### 2.4.3 GIoU loss function

The boundary box loss function is an important component of the object detection loss function. A well-defined boundary box loss function can significantly improve the performance of object detection models. In YOLOv11, CIoU is used as the regression box loss function. Although CIoU improves upon GIoU by introducing center distance and aspect ratio constraints, the additional constraints introduced by CIoU might lead to overfitting or convergence difficulties in orchard data collection, where there is a large variation in target size (due to close and distant objects) and where the aspect ratio differences of pear fruit bounding boxes are not significant. Moreover, compared to GIoU, the calculation of the aspect ratio parameter *v* in CIoU is relatively more complex (Zheng et al., 2020), resulting in higher computational costs during training and slower model convergence. Therefore, this study replaces CIoU with the GIoU loss function. The GIoU loss function is used in object detection to measure the difference between the predicted and ground truth boxes, addressing the issue where traditional IoU fails to provide effective gradient feedback when the predicted box and the ground truth box do not overlap. This improves the model’s convergence and accuracy. GIoU loss not only considers the overlapping region between boxes but also takes into account the spatial relationship between the boxes by introducing the concept of the minimal enclosing box. This allows the model to learn the shape and position of the boxes more accurately, ultimately enhancing the performance of object detection.

### 2.5 Experimental environment and parameter settings

The experimental environment for this study runs on the Windows 10 operating system, equipped with 32 GB of memory and an NVIDIA GeForce RTX 4080 GPU, with an Intel(R) Core(TM) i7-13700F @2.10GHz processor. The deep learning framework used is PyTorch 2.0.1, with CUDA 11.8 and CUDNN 8.8.0.

The network training parameters are set as follows: The image input size is 640 × 640, and the batch size is set to 32; the maximum number of iterations is 200. The optimizer is SGD, with the learning rate dynamically adjusted using a cosine annealing strategy. The initial learning rate is set to 0.01, the momentum factor is 0.937, and the weight decay coefficient is 0.0005.

### 2.6 Evaluation metrics

Object detection models should be evaluated using multiple metrics to provide a comprehensive assessment of their performance. To evaluate the performance of ADG-YOLO, seven metrics are used: precision, recall, average precision (AP), mean average precision (mAP), number of parameters, model size, and GFLOPs. These metrics offer a well-rounded evaluation of ADG-YOLO’s performance in the multi-category pear fruit detection task within the complex environment of a pear orchard. They reflect the model’s performance across various dimensions, including accuracy, recall, speed, and efficiency. The formulas for calculating the relevant performance metrics are provided, as shown in Equations 1-4.


(1) $$\text{P}=\frac{TP}{TP+FP}$$

(2) $$\text{R}=\frac{TP}{TP+FN}$$

(3) $$\text{AP}=\int_{0}^{1}\text{P}\left(\text{R}\right)\text{dR}$$

(4) $$\text{mAP}=\frac{\sum_{i=1}^{C}AP_{i}}{C}$$

Where TP represents the number of true positive samples that the model correctly predicted as positive, FP represents the number of false positive samples that the model incorrectly predicted as positive, and FN represents the number of false negative samples that the model incorrectly predicted as negative. AP refers to the area under the Precision-Recall (P-R) curve, while mAP refers to the mean value of the AP for each class.

## 3 Results

### 3.1 Ablation experiment

To evaluate the effectiveness and feasibility of the proposed AHG-YOLO model in detecting pear fruits with no occlusion, partial occlusion, and fruit overlap, an ablation experiment was conducted based on the YOLOv11n model. Each improvement method and the combination of two improvement methods were added to the YOLOv11n model and compared with the AHG-YOLO model. In the experiment, the hardware environment and parameter settings used for training all models remained consistent. **Table 2** shows the ablation experiment results of the improved YOLOv11n model and the AHG-YOLO model on the test set. After introducing the ADown downsampling module to enhance the feature extraction capability of the YOLOv11 network, the model’s precision, recall, AP, and mAP@0.5:0.95 increased by 2.2%, 2.6%, 1.8%, and 2.1%, respectively. The model’s parameter count decreased by 18.6%, GFLOPs decreased by 15.9%, and model size decreased by 17.3%. This indicates that the ADown module can effectively improve the pear object detection accuracy. After introducing the EfficientHead detection head, although the model’s precision, recall, and AP decreased slightly, mAP@0.5:0.95 increased by 0.1%, the model’s parameter count reduced by 10.4%, GFLOPs reduced by 19.0%, and model size decreased by 9.62%. This suggests that EfficientHead plays a significant role in model lightweighting. As shown in **Table 2**, after introducing the ADown module and GIoU, although the model’s parameter count increased, precision, recall, mAP@0.5, and mAP@0.5:0.95 increased by 1.5%, 1.7%, 0.4%, and 1.6%, respectively. After introducing the ADown module and EfficientHead, precision, recall, mAP@0.5, and mAP@0.5:0.95 increased by 2.3%, 2.2%, 1.7%, and 2.5%, and the model’s parameter count, GFLOPs, and model size all decreased. Additionally, after introducing EfficientHead and GIoU, recall, mAP@0.5, and mAP@0.5:0.95 all increased compared to their individual introduction, without increasing the parameter count. Finally, the proposed AHG-YOLO network model outperforms the original YOLOv11 model, with precision, recall, mAP@0.5, and mAP@0.5:0.95 improving by 2.5%, 3.6%, 2.3%, and 2.6%, respectively. Meanwhile, GFLOPs are reduced to just 4.7, marking a 25.4% decrease compared to the original YOLOv11n, the parameter count decreased by 16.9%, and the model size is only 5.1MB.

**Table 2:** Comparison of ablation experiment results between the improved YOLOv11n and AHG-YOLO.

Methods	ADown	EfficientHead	GIoU	P/%	R/%	mAP@0.5/%	mAP@0.5:0.95/%	Params	GFLOPs	Model size(M)
YOLOv11n				88.8	84.5	91.8	69.2	258,2737	6.3	5.2
YOLOv11-A	✓			91	87.1	93.6	71.3	210,3505	5.3	4.3
YOLOv11-H		✓		88.2	83.3	90.7	69.3	231,3041	5.1	4.7
YOLOv11-G			✓	86.1	85.3	90.8	69	258,2737	6.3	5.2
YOLOv11-AG	✓		✓	90.3	86.2	92.2	70.8	294,1905	6.0	5.9
YOLOv11-AH	✓	✓		91.1	86.7	93.5	71.7	253,9089	4.7	5.1
YOLOv11-HG		✓	✓	88	85.5	91.4	69.6	231,3041	5.1	4.7
AHG-YOLO	✓	✓	✓	91.3	88.1	94.1	71.8	253,9089	4.7	5.1

H stands for the improved method using EfficientHead, and G represents replacing CIoU with GIoU.

According to the data in **Table 2**, the mAP@0.5 of YOLOv11-A reached 93.6%, an improvement over the baseline model YOLOv11n. However, when H or G were added individually, the mAP@0.5 dropped to 90.7% and 90.8%, respectively. When combined with the A module, the mAP values increased again. The reasons for this can be analyzed as follows: The ADown module significantly improves baseline performance by preserving discriminative multi-scale features through adaptive downsampling. The EfficientHead method reduces model parameters and computational load compared to the baseline model, but the simplified model structure leads to information loss and a decrease in detection accuracy. GIoU performs poorly on bounding box localization in raw feature maps, resulting in a drop in detection accuracy. When combined with ADown, the ADown module optimizes the features, providing better input for the subsequent EfficientHead and GIoU, thus leveraging the complementary advantages between the modules. The optimized features from ADown reduce the spatial degradation caused by EfficientHead, maintaining a mAP@0.5 of 93.5%, while reducing GFLOPs by 11.3%. ADown’s noise suppression allows GIoU to focus on key geometric deviations, improving localization robustness. The synergy of all three modules achieves the best accuracy-efficiency balance (94.1% mAP@0.5, 4.7 GFLOPs), where ADown filters low-level redundancies, EfficientHead enhances discriminative feature aggregation, and GIoU refines boundary precision. This analysis shows that H and G are not standalone solutions, they require the preprocessing from ADown to maximize their effectiveness.

**Figure 9:**
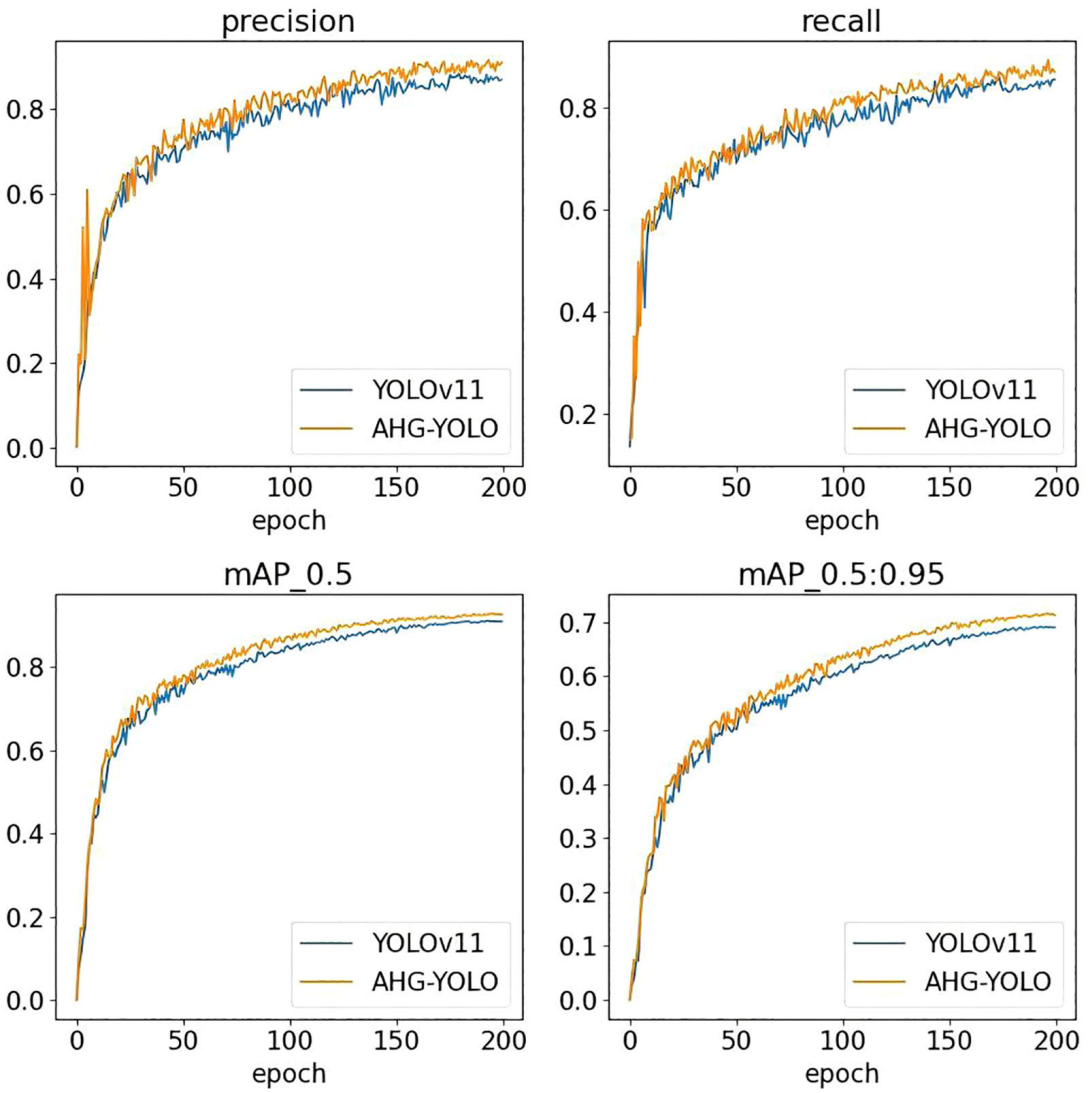
Comparison of detection accuracy between AHG-YOLO and YOLOv11n.

**Figure 10:**
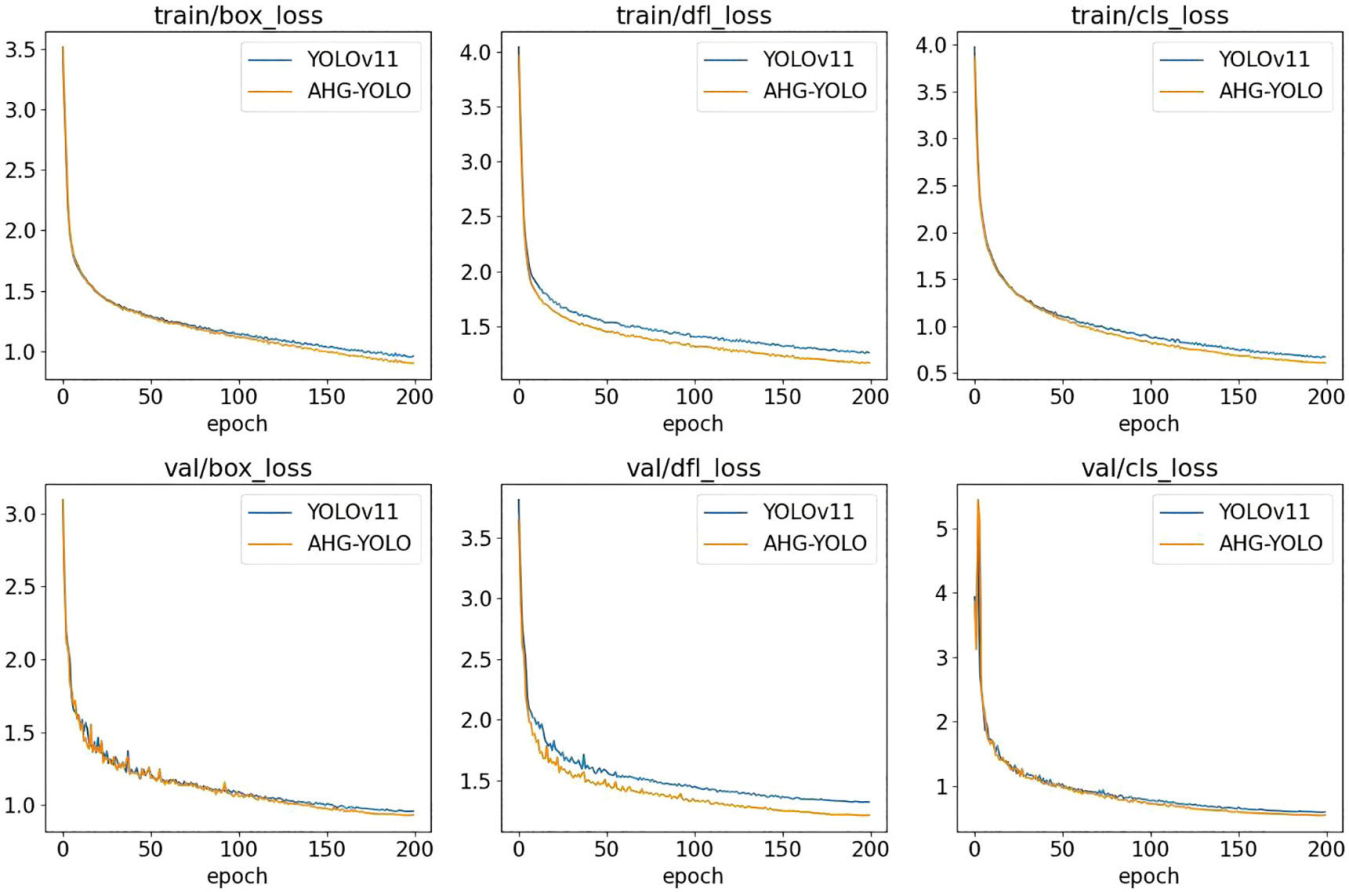
Comparison of loss values between AHG-YOLO and YOLOv11n.

**Figures 9** and 10 show the performance of AHG-YOLO compared to YOLOv11n during the training process. From **Figures 9**, **10**, it can be seen that during 200 training iterations, the proposed AHG-YOLO achieves higher detection accuracy and obtains lower loss values compared to YOLOv11n. This indicates that the AHG-YOLO network model can effectively improve the detection accuracy of pears in unstructured environments and reduce the false detection rate.

**Figure 11:**
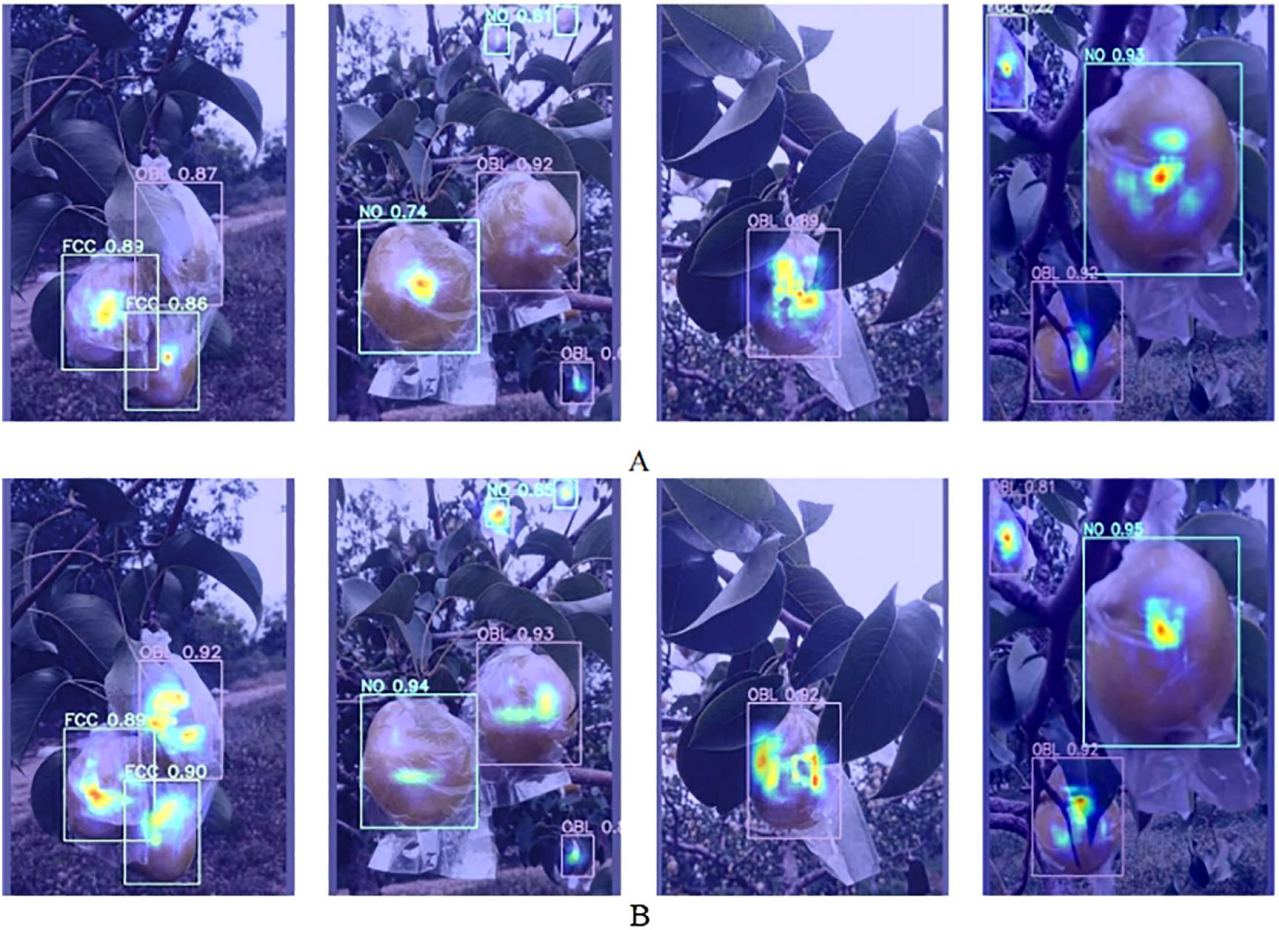
Heatmap of YOLOv11n and AHG-YOLO. **(A)** YOLOv11n; **(B)** AHG-YOLO.

The Grad-CAM (Selvaraju et al., 2016) method is used to generate heatmaps to compare the feature extraction capabilities of the YOLOv11n model and the AHG-YOLO model in complex scenarios such as overlapping fruits, small target fruits, and fruit occlusion, as shown in **Figure 11**. **Figure 11** shows that the AHG-YOLO model exhibits better performance in complex scenarios. The specific quantitative results comparison can be found in Section 3.3.

**Figure 12:**
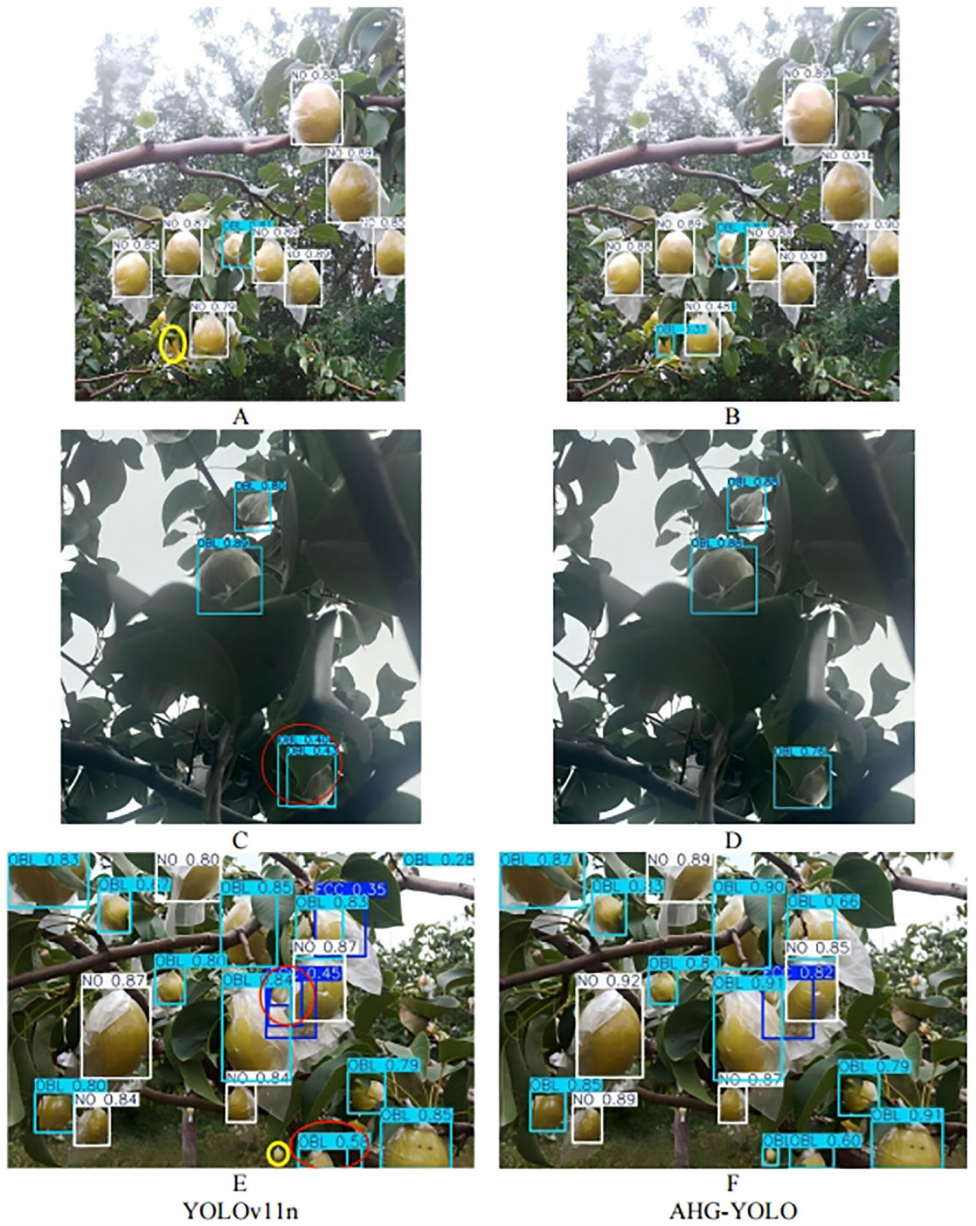
**(A)** YOLOv11n detection results for long-distance images. **(B)** AHG-YOLO detection results for long-distance images. **(C)** YOLOv11n detection results under low light conditions. **(D)** AHG-YOLO detection results under low light conditions. **(E)** YOLOv11n detection results in dense fruit scenarios. **(F)** AHG-YOLO detection results in dense fruit scenarios.

To further validate the detection performance, experiments were conducted on the test dataset for both the YOLOv11n model and the AHG-YOLO model. The detection results are shown in **Figure 12**, where the red circles represent duplicate detections and the yellow circles represent missed detections. By comparing **Figures 12A, B**, it can be observed that YOLOv11n has one missed detections. By comparing **Figures 12C, D**, it can be seen that YOLOv11n has one duplicate detections. By comparing **Figures 12E, F**, it can be seen that YOLOv11n has two duplicate detections and one missed detections. This demonstrates that AHG-YOLO can accurately perform multi-class small object detection and classification in complex environments, exhibiting high accuracy and robustness, and effectively solving the pear detection problem in various scenarios within complex environments.

### 3.2 Detection results of pear targets in different classes

**Figure 13:**
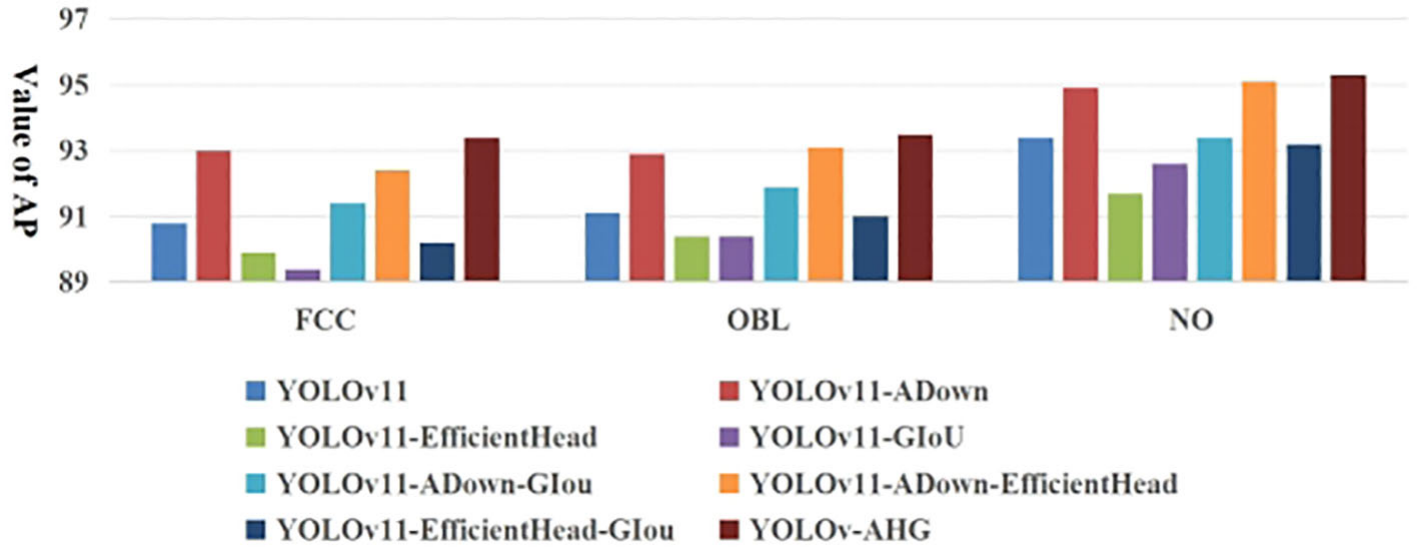
AP results of detecting different types of pear targets with different networks.

**Figure 13** shows the AP results for multi-category detection for occluded pear fruits in complex orchard scenes by different networks on the test set. **Table 3** presents the specific detection results of AHG-YOLO and YOLOv11n for different categories of pear targets on the test set. From **Figure 13** and **Table 3**, it can be observed that the base YOLOv11 network performs best in detecting NO fruit, with an AP value of 93.4%, but performs relatively poorly when detecting FCC and OBL fruits. The proposed AHG-YOLO model improves the AP for detecting FCC fruits by 2.6%, reaching 93.4%, improves the AP for detecting OBL fruits by 2.4%, reaching 93.5%, and improves the AP for detecting NO fruits by 1.9%, reaching 95.3%. This indicates that the proposed method is highly effective for fruit target detection in complex environments, demonstrating both excellent accuracy and robustness.

**Table 3:** Detection results of different types of pear targets by AHG-YOLO and YOLOv11n.

Model	Categories	P/%	R/%	mAP@0.5/%	mAP@0.5:0.95/%
YOLOv11n	All	88.8	84.5	91.8	69.2
	FCC	89.6	80.9	90.8	64.8
	OBL	89.3	83.3	91.1	65.6
	NO	87.5	89.2	93.4	77.2
AHG-YOLO	All	91.3	88.1	94.1	71.8
	FCC	92.1	85.5	93.4	67.9
	OBL	90.5	87.6	93.5	68.8
	NO	91.3	91.2	95.3	78.7

### 3.3 Comparison with mainstream object detection models

AHG-YOLO was compared with other mainstream object detection networks, and the detection results on the test set are shown in **Table 4**. The experimental results of all models indicate that YOLOv9c achieves the highest precision, mAP@0.5, and mAP@0.5:0.95 among all models. However, the YOLOv9c model has excessively large parameters, GFLOPs, and model size, making it unsuitable for real-time detection in harvesting robots. AHG-YOLO’s mAP@0.5 surpasses that of Faster R-CNN, RTDETR, YOLOv3, YOLOv5n, YOLOv7, YOLOv8n, YOLOv10n, and YOLOv11n by 15.1%, 0.9%, 2.4%, 3.9%, 12.6%, 3.4%, 5.2%, and 2.3%, respectively. In terms of precision, recall, mAP@0.5:0.95, and GFLOPs, AHG-YOLO also shows advantages. Therefore, based on a comprehensive comparison of all metrics, AHG-YOLO is better suited for pear target detection tasks in complex environments.

**Table 4:** Comparison of results from different object detection models.

Model	P/%	R/%	mAP@0.5/%	mAP@0.5:0.95/%	Params (M)	GFLOPs	Model size (M)
Faster R-CNN	77.03	71.7	79.0	54.8	136.73	369.768	108
RTDETR	93.0	90.7	93.2	71.9	28.45	100.6	336
YOLOv3	87.8	84.6	91.7	71.7	98.48	262.6	752
YOLOv5n	89	82.1	90.2	66.5	2.18	5.8	4.6
YOLOv7	78.6	74.1	81.5	52	6.02	13.2	12.3
YOLOv8n	87.9	84.2	90.7	68.2	2.68	6.8	5.6
YOLOv9c	93.4	90.4	95.4	76.5	21.15	82.7	41.2
YOLOv10n	86.2	81.3	88.9	66.7	2.27	6.5	5.8
YOLOv11n	88.8	84.5	91.8	69.2	2.58	6.3	5.2
AHG-YOLO	91.3	88.1	94.1	71.8	2.54	4.7	5.1

## 4 Discussion

YOLO series detection algorithms are widely used in fruit detection due to their high detection accuracy and fast detection speed. These algorithms have been applied to various fruits, such as tomatoes (Wu H, et al., 2024), kiwifruits (Yang et al., 2024), apples (Wu M, et al., 2024), achieving notable results. Researchers have always been focused on designing lightweight algorithms, and this is also true for pear fruit target detection. Tang et al. (2024) proposed a pear target detection method based on an improved YOLOv8n for fragrant pears. Using their self-built fragrant pear dataset, they improved the F0.5-score and mAP by 0.4 and 0.5 percentage points compared to the original model, reaching 94.7% and 88.3%, respectively. Li et al. (2022) introduced the advanced multi-scale collaborative perception network YOLOv5sFP for pears detection, achieving an AP of 96.12% and a model size of 50.01 MB.

While these studies have achieved remarkable results, they did not address the practical needs of robotic harvesting, as they focused solely on detecting a single class of pear fruits. This study takes into account the detection requirements for robotic harvesting, categorizing pear fruits in orchards into three groups (ON, OBL, FCC) to enable the harvesting robot to develop different harvesting strategies based on conditions of no occlusion, branch and leaf occlusion, and fruit overlap, thus improving harvesting efficiency. Compared to commonly used detection models, the AHG-YOLO proposed in this study achieves the highest detection accuracy in complex orchard environments, with an mAP of 94.1%.

**Figure 14:**
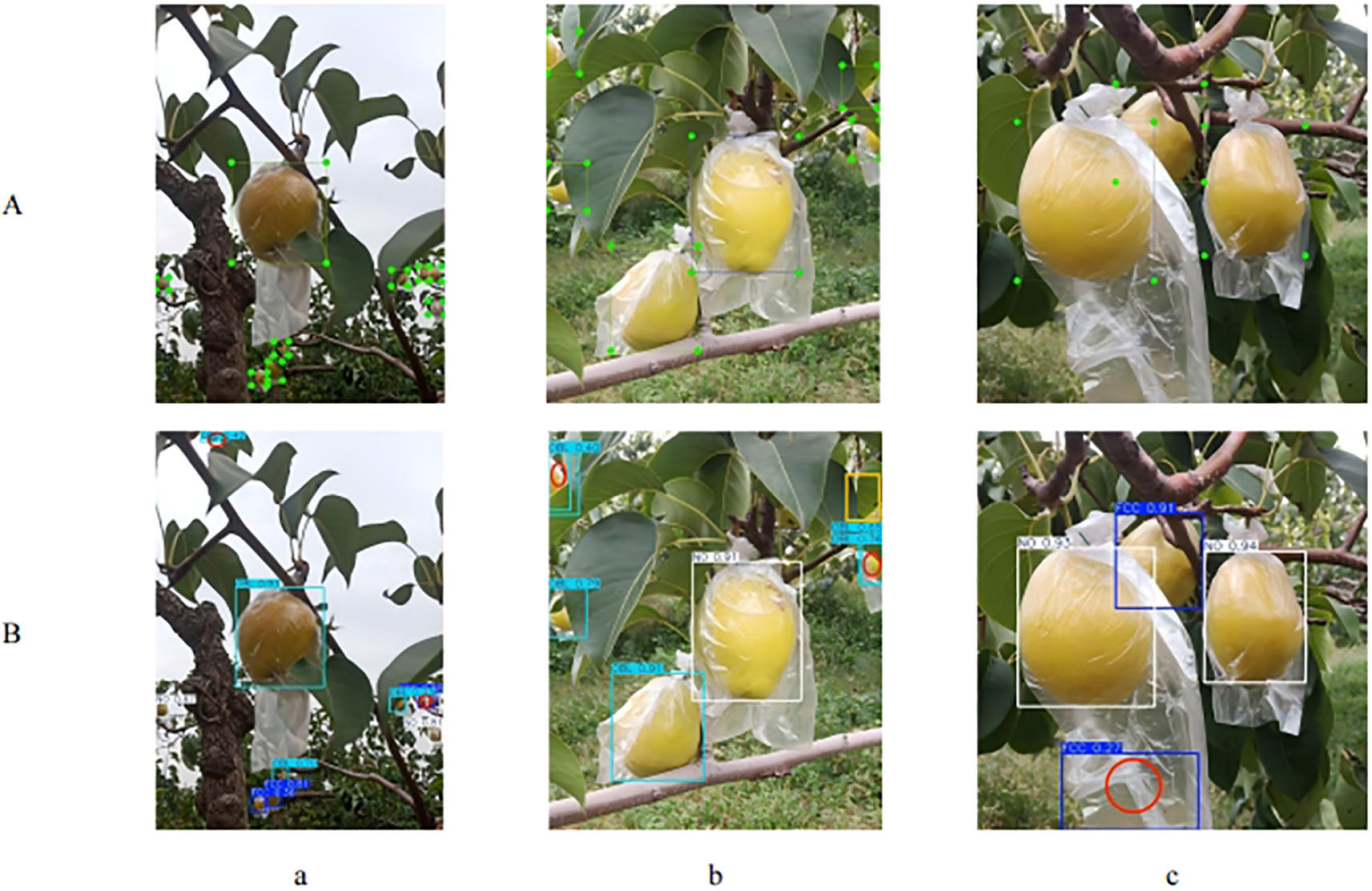
Three example images with detection errors are shown, demonstrating the multi-class pear fruit detection using AHG-YOLO. **(A)** Manually annotated image; **(B)** AHG-YOLO **(a-c)** numbered example images. Each position marked with a red circle indicates a detection error of the pear fruit, while each position marked with a yellow square indicates a missed detection.

**Figure 14** shows three examples of detection errors when using AHG-YOLO for multi-category detection of occluded pear fruits. The potential causes of these errors are as follows: (1) In cloudy, dim lighting conditions, when fruits are tightly clustered and located at a distance, the fruit targets appear small, making feature extraction challenging. This leads to repeated detection of FCC fruits, as seen in the lower right red circle of **Figure 14A**. Additionally, the dim lighting causes the occluded pear’s features to resemble those of the leaves, resulting in the model mistakenly detecting leaves as OBL fruits, as shown in the upper left red circle of **Figure 14A**. (2) When the target is severely occluded, the model struggles with feature extraction, which may lead to either missed detections or repeated detection, as shown in **Figure 14B**. The yellow bounding box indicates a missed detection, and the red circle indicates a repeated detection. (3) Detecting FCC fruits is particularly challenging because the fruits are often clustered together, making it difficult to distinguish between them. Furthermore, the fruit bags sometimes interfere with the detection process, causing errors, as seen in **Figure 14C**, where the bag is incorrectly detected as an FCC fruit.

To enhance the accuracy of AHG-YOLO in detecting multi-category detection for occluded pear fruits, the following measures can be taken: (1) Increase the number of samples that are prone to detection errors, such as FCC and OBL class samples, to diversify the dataset and improve the model’s detection capability in complex environments. (2) Further refine the model’s feature extraction capability, particularly for detecting small targets.

Although the AHG-YOLO model has some limitations in detecting multi-category detection for occluded pear fruits, it achieves an overall detection mAP of 94.1%, which meets the fruit detection accuracy requirements for orchard automation in harvesting. This provides crucial technical support for robotic pear harvesting in orchards. The AHG-YOLO model will be applied to the visual detection system of pear fruit-picking robots to validate its reliability.

## 5 Conclusion

This paper proposes the AHG-YOLO network model for multi-category detection of occluded pear fruits in complex orchard scenes. Using YOLOv11n as the base model, the ADown downsampling method, lightweight detection head, and GIoU loss function are integrated to enhance the network’s feature extraction capability and reduce the model’s complexity, making it suitable for real-time harvesting applications. The conclusions are as follows:

(1) Experimental results in complex pear orchard environments demonstrate that the mAP of AHG-YOLO for multi-category detection for occluded pear fruits is 94.1%, with the AP for FCC, OBL, and NO fruits being 93.4%, 93.5%, and 95.3%, respectively. Compared to the base YOLOv11n network, precision, recall, mAP@0.5, and mAP@0.5:0.95 improved by 2.5%, 3.6%, 2.3%, and 2.6%, respectively. Additionally, GFLOPs are reduced to 4.7, representing a 25.4% decrease compared to the original YOLOv11n, while the number of parameters is reduced by 16.9%, and the model size is just 5.1MB.

(2) Compared with eight other commonly used object detection methods, AHG-YOLO achieves the highest detection accuracy while maintaining a lightweight design. The mAP@0.5 is 15.1%, 0.9%, 2.4%, 3.9%, 12.6%, 3.4%, 5.2%, and 2.3% higher than Faster R-CNN, RTDETR, YOLOv3, YOLOv5n, YOLOv7, YOLOv8n, YOLOv10n, and YOLOv11n, respectively, thereby meeting the real-time harvesting requirements of orchards.

In summary, the AHG-YOLO model proposed in this paper provides a solid methodological foundation for real-time pear target detection in orchard environments and supports the development of pear-picking robots. Future work will focus on further validating the effectiveness of the method in pear orchard harvesting robots, with ongoing optimization efforts.

## Data Availability

The original contributions presented in the study are included in the article/supplementary material. Further inquiries can be directed to the corresponding author.
